# Supplementary material for: An adhesion G protein-coupled receptor is required in cartilaginous and dense connective tissues to maintain spine alignment
Source: eLife. 2021 Jul 28;10:e67781. doi: 10.7554/eLife.67781 (PMC8328515; doi:10.7554/eLife.67781)
Supplement: Supplementary file 1. — Data shows µg estimated for total reaction volume; w/w percentage. CS: chondroitin sulfate; HA: hyaluronan; GAG: glycosaminoglycan; ND: not detected. [file elife-67781-supp1.docx]

Supplementary File 1: Chondroitin sulfate digestion profile and total hyaluronan content in Cre (-) control and *Col2a1-Cre; Adgrg6^f/f^* mutant mice at P20

|  | **Control-1** | | **Control-2** | | **Control-3** | | **Mutant-1** | | **Mutant-2** | |
| --- | --- | --- | --- | --- | --- | --- | --- | --- | --- | --- |
| **CS** |  |  |  |  |  |  |  |  |  |  |
| D0a0 | 3.88 | *4.0* | 6.10 | *4.6* | 7.35 | *3.8* | 6.30 | *5.4* | 4.45 | *5.1* |
| D0a6 | 9.34 | *9.5* | 11.31 | *8.5* | 15.96 | *8.3* | 9.56 | *8.1* | 6.99 | *8.0* |
| D0a4 | 83.25 | *85.1* | 113.92 | *85.5* | 167.09 | *87.0* | 100.49 | *85.4* | 75.05 | *86.1* |
| D2a0 | ND |  | ND |  | ND |  | ND |  | ND |  |
| D2a6 | ND |  | ND |  | ND |  | ND |  | ND |  |
| D0a10 | 0.25 | *0.3* | 0.63 | *0.5* | 0.34 | *0.2* | 0.30 | *0.3* | 0.14 | *0.2* |
| D2a4 | 1.12 | *1.1* | 1.30 | *1.0* | 1.19 | *0.6* | 1.09 | *0.9* | 0.59 | *0.7* |
| D2a12 | ND |  | ND |  | ND |  | ND |  | ND |  |
| Total CS | 97.84 | *100* | 133.26 | *100* | 191.92 | *100* | 117.73 | *100* | 87.22 | *100* |
| Total HA | 4.09 |  | 3.41 |  | 1.44 |  | 5.41 |  | 2.05 |  |
| Total GAG | 101.93 |  | 136.67 |  | 193.36 |  | 123.14 |  | 89.27 |  |
